# Pathological Conditions Associated with the Male Reproductive Tract of the Sahel Bucks

**DOI:** 10.1155/2014/406431

**Published:** 2014-03-24

**Authors:** Yusuf Abba, Suleiman Simon, Halima Idris Gambo, Ikechukwu Onyebuchi Igbokwe, Yusuf Iliyasu

**Affiliations:** ^1^Department of Veterinary Pathology, Faculty of Veterinary Medicine, University of Maiduguri, PMB 1069, Maiduguri, Borno State, Nigeria; ^2^Department of Veterinary Pathology and Microbiology, Faculty of Veterinary Medicine, Universiti Putra Malaysia, 43400 Serdang, Selangor, Malaysia

## Abstract

The study of pathological conditions of the male reproductive system is paramount to understanding reproductive inefficiency in the Sahel goat. In this study, 1048 Sahel bucks presented for slaughter at the Maiduguri metropolitan abattoir were evaluated for the presence of various pathological abnormalities of the reproductive system. A total incidence of 15.08% was recorded for various pathological conditions, with testicular, penile, and scrotal conditions having incidences of 7.82%, 4.80 and 2.50%, respectively. Bilateral testicular hypoplasia and atrophy and unilateral cryptorchidism accounted for incidences of 4.10%, 2.38%, and 1.24%, respectively, while paraphimosis and scrotal laceration had incidences of 1.72% and 1.05%, respectively. Age specific incidence of pathological conditions were not significant (*P* > 0.05) between bucks aged <1–1.5 and 2–2.5 years. However, bucks aged 3–3.5 year a had lower (*P* < 0.05) incidence of pathological conditions than other age groups. Histopathological evidence of inflammation, degeneration, and atrophy was observed in the testes, while inflammatory changes were observed in the prepuce.

## 1. Introduction

The male reproductive system is essential for domestic animals' species propagation and survival [1]. The testis and epididymis are important components of this system responsible for spermatogenesis and transfer of sperm cells into the vas deferens, while the penis plays an important role in copulation and subsequent ejaculation of semen into the female genitalia [2]. Pathological conditions affecting the male reproductive system have been reported to affect the function of the system and predispose to the development of infertility or sterility in farm animals [3]. The occurrence of testicular pathologies such as hypoplasia, degeneration, atrophy, and cryptorchidism has been previously reported in the Sahel goat [4–6] as well as in other goat breeds worldwide [7–9]. However, there is still paucity of information on the incidence of nontesticular pathological conditions associated with the penis and scrotum of the Sahel goat. Therefore, this study was undertaken in order to evaluate the incidence of different pathological conditions associated with the reproductive tract of the male Sahel goat in northeastern Nigeria.

## 2. Materials and Methods

One thousand and forty-eight male Sahel goats presented for slaughter at the Maiduguri metropolitan abattoir in March–July, 2010, were used for this study. The age of the bucks was estimated before slaughter by dental examination. The bucks were categorized into three age groups of <1–1.5, 2–2.5, and 3–3.5 years. After slaughter, the whole genitalia consisting of the penis, prepuce, testes, epididymis, and scrotal sac were examined for evidence of gross pathological abnormalities. Testicular conditions such as bilateral testicular hypoplasia and atrophy were identified through gross changes in size, texture, and consistency of the testes. Briefly, hypoplastic testes were identified as small, soft, and flabby, while atrophic testes were small and hard due to regression in size and thickening of the tunica albuginea. Unilateral cryptorchidism was identified on the basis of retention of one testicle (right of left) either in the abdominal or inguinal cavities. Tissue samples were collected and fixed in 10% buffered formalin, processed, sectioned, and stained with hematoxylin and eosin for histopathological examination using light microscopy. Confirmation of testicular hypoplasia and atrophy was done microscopically by examination of the seminiferous tubules from testicular tissue sections for evidence of spermatogenesis, degeneration, inflammation, and fibrosis. Data obtained were summarized and presented as number of occurrences and percent or proportion of total population examined. A statistical comparison among the age groups was made with *Z*-test using computer statistical software (JMP 9, QSAS Institute Inc, Cary, NC, USA).

## 3. Results

**Figure 1:**
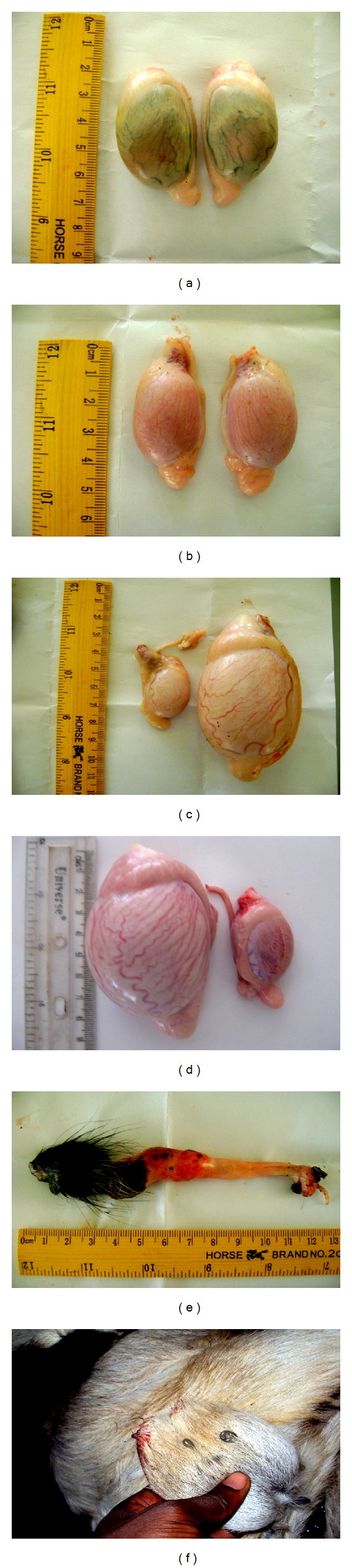
Male reproductive system of the Sahel goat showing (a) bilateral testicular atrophy, (b) bilateral testicular hypoplasia, (c) unilateral cryptorchidism, (d) unilateral testicular hypoplasia, (e) balanitis, and (f) ectoparasites (ticks) on the scrotal skin.

Evidence of some gross lesions observed in the testes, penis, and scrotum is shown in Figure 1. A total incidence of 15.08% was observed for various pathological conditions associated with the male reproductive system of the Sahel goat. Out of this total, 7.82% were testicular and 4.80% were penile, while 2.48% were scrotal related conditions, representing 51.90%, 31.60%, and 16.40% of the total incidence in the population. Bilateral testicular hypoplasia (BTH) accounted for the highest incidence of 4.10%, while bilateral testicular atrophy (BTA), paraphimosis, and unilateral cryptorchidism (UC) followed with incidence rates of 2.38%, 1.72%, and 1.24%, respectively (Table 1). Other pathological conditions observed in the male reproductive system were balanitis, balanoposthitis, posthitis, penile laceration, scrotal laceration, scrotal ectoparasitism and hydrocoele (Table 1).

The age specific incidence of pathological conditions in the male reproductive tract of the Sahel goat is summarized in Table 2.

The age specific incidence of all pathological conditions encountered was 18.85% in bucks aged 2–2.5 years, 15.06% in bucks aged <1–1.5 years, and 8.46% in bucks aged 3–3.5 years. These incidences did not vary significantly (*P* > 0.05) between bucks aged <1–1.5 years and 2–2.5 years. Similarly, there was no significant difference (*P* > 0.05) between all testicular and penile conditions observed in bucks aged <1–1.5 years and 2–2.5 years. However, there was a significant difference (*P* < 0.05) in the occurrence of scrotal conditions in these two age groups. In bucks aged 3–3.5 years, BTH, UC, and balanoposthitis had a significantly lower (*P* < 0.05) incidence when compared with incidences in bucks aged <1–1.5 and 2–2.5 years. The incidence of all other conditions did not vary significantly (*P* > 0.05) among the three age groups.

**Figure 2:**
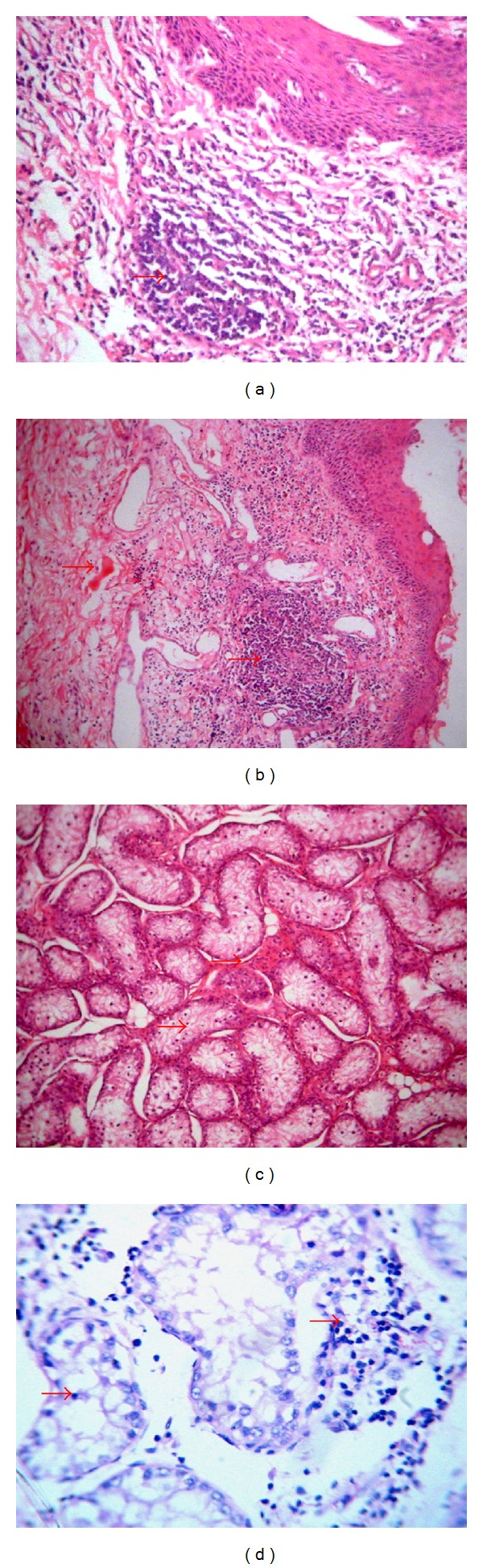
Histological section of the (a) preputial skin, showing marked leucocytic infiltration in inflammatory response in the dermis, H&E ×100, (b) preputial skin, showing hyperemia in the hypodermis and marked leucocytic infiltration in inflammatory response in the dermis, H&E ×40, (c) testis showing seminiferous tubules devoid of spermatogenic cell developmental stages, H&E ×100, and (d) testis showing atrophic seminiferous tubules with degenerate spermatocytes and inflammatory response in the interstitium, H&E ×200.

Histopathological evidence of suppurative inflammatory responses marked by neutrophil and monocyte infiltration was observed in the prepuce and preputial skin in posthitis and balanoposthitis. In testicular hypoplasia and cryptorchidism, the seminiferous tubules had reduced luminal diameters with complete absence of spermatogenic cell maturation stages. In testicular atrophy, there were degenerate spermatogenic cells within the lumen of the seminiferous tubules, accompanied with or without an interstitial inflammatory response and fibrosis (Figure 2).

## 4. Discussion

An incidence of 5.26% for testicular lesions reported in Algerian bucks [9] was comparatively lower than what was reported in this study. In a related study [10], the total genital abnormality observed in bucks was 17.80%. This value is slightly higher than what was observed in this study, despite the fact that the sample size used in the study was considerably lower than that of this study.

The prevalence of bilateral testicular hypoplasia previously reported in Sahel bucks aged 1.9 ± 0.3 years was 1.4% [5]. In this study, we observed a much higher incidence of 5.48% and 5.42% in bucks aged 2–2.5 and <1–1.5 years. This study agreed with the previous work by [5], in which BTH was most frequently observed in Sahel bucks within the age range of 2–2.5 years. In a related study of slaughtered bucks in Algeria, the incidence of BTH was reported as 0.70%, while that of unilateral testicular hypoplasia (UTH) was 1.4% [9]. Another study [8] reported a prevalence of 6.4% for testicular degeneration or hypoplasia in Iranian bucks. The study reported that bucks aged >4 years had the highest age specific prevalence of 16.4%, while bucks aged 2-3 years had a prevalence of 11.6%. These values are much higher than what was observed in this study.

The prevalence of BTA in the Sahel buck was reported as 0.08% [6], which is lower than the value reported in this study (2.38%). However, a related study [10] reported a total prevalence of 9.7% for BTA in bucks slaughtered in Ethiopia. The authors observed an age specific prevalence of 6.6%, 11.8%, and 25% in bucks aged <1, 1, and 2 years of age, respectively. These values are higher and in contrast with what was observed in this study, where age specific incidences of BTA were 1.81%, 3.29%, and 1.54% in bucks aged <1–1.5, 2–2.5, and 3–3.5 years, respectively.

The total incidence of UC in this study was 1.24%, and this is twice the prevalence rate (0.6%) reported for the Sahel buck from a previous study [4]. In other related studies, the prevalence of UC in bucks in Algeria, Iran, and Ethiopia was 2.1%, 2.9%, and 5.5%, respectively [8–10]. All these rates are higher than what was observed in this study and from a previous study [4]. Although we only observed UC in bucks aged <1–1.5 (1.20%) and 2–2.5 (1.97%) years, other authors [8] reported UC across all age groups, with the highest prevalence in bucks >4 years (8.2%). Similarly, age specific prevalence of UC was reported as 4.6% and 6.7% in bucks aged <1 and 1 years, respectively [10].

Balanoposthitis had an incidence of 0.86%; this was slightly lower than the prevalence reported for ulcerative balanoposthitis in feral male goats (1.1%) [7]. Other genital conditions that were observed in the penis, prepuce, and scrotum have not been previously reported in the Sahel goats. However, commonly observed inflammatory and degenerative conditions of the genital organs such as orchitis, epididymitis, sperm granuloma, and mineralization, which were reported by several authors in other goat breeds, were not encountered during the course of this study [8–10].

The histopathological evaluation of testicular conditions (BTH, BTA, and UC) showed findings that were consistent with what was reported in the literature [3]. Bucks with BTH and UC had hypoplastic seminiferous tubules that were mostly devoid of spermatogenic cells, while those with BTA had degenerate and fibrotic seminiferous tubules that were mostly accompanied by an inflammatory response in the interstitium. These findings have been previously reported in the Sahel goats [4, 5], as well as in other goat breeds [8–10].

UC and BTH are congenital conditions that are hereditary and irreversible, while BTA is mostly acquired due to chronic pathologic changes affecting the testes such as degeneration and inflammation [4–6]. In this study 75.2% of the bucks presented for slaughter were below the age of 3 years, while only 24.8% were above 3 years. Previously, [6] attributed increased culling of young Sahel bucks as a reason for low prevalence of BTA in mature Sahel bucks. Similarly, a higher prevalence of BTH was observed in bucks aged 2–2.5 years than in older bucks [5, 10]. On the contrary, [8] reported a high prevalence of BTH (16.4%) and UC (8.2%) in bucks aged >4 years. Since no investigation was conducted to ascertain the reason for the variation in the incidences among the young and old age groups, we cannot categorically attribute a specific factor to be responsible for the low incidence of these testicular conditions in older Sahel bucks. However, we presume that since these conditions are known to affect reproductive efficiency, most farmers notice it early and sell the bucks at a younger age, thus, resulting in a lower incidence in older bucks.

## 5. Conclusion

It was observed that testicular conditions were more common in occurrence than penile or scrotal conditions in the male Sahel goats. Similarly, it was also noted that bucks aged <1–1.5 and 2–2.5 years had higher incidence of all genital conditions than those aged 3–3.5 years.

## Figures and Tables

**Table 1:** Incidence of pathological conditions encountered in the male reproductive tracts of Sahel goats.

Reproductive tract pathologies	Number (%) in population
Testicular conditions	
Bilateral testicular hypoplasia	43 (4.10)
Bilateral testicular atrophy	25 (2.38)
Unilateral cryptorchidism	13 (1.24)
Unilateral testicular hypoplasia	1 (0.10)
Penile conditions	
Balanitis	5 (0.48)
Posthitis	9 (0.86)
Balanoposthitis	9 (0.86)
Paraphimosis	18 (1.72)
Penile laceration	9 (0.86)
Scrotal conditions	
Scrotal laceration	11 (1.05)
Scrotal ectoparasitism	4 (0.38)
Hydrocoele	5 (0.48)
Hydrocoele with scrotal ectoparasitism	1 (0.10)
Scrotal laceration with posthitis	1 (0.10)
Scrotal laceration with balanitis	4 (0.38)

Total	158 (15.08)

**Table 2:** Age specific incidence of pathological conditions in the male reproductive tracts of Sahel goats.

Reproductive tract pathologies	Number (%) in population based on age		
	<1–1.5	2–2.5	3–3.5
No of bucks examined	**332**	**456**	**260**
Testicular conditions			
Bilateral testicular hypoplasia	18 (5.42)^a^	25 (5.48)^a^	0 (0)^b^
Bilateral testicular atrophy	6 (1.81)^a^	15 (3.29)^a^	4 (1.54)^a^
Unilateral cryptorchidism	4 (1.2)^a^	9 (1.97)^a^	0 (0)^b^
Unilateral testicular hypoplasia	0 (0)^a^	0 (0)^a^	1 (0.38)^a^
Penile conditions			
Balanitis	2 (0.6)^a^	2 (0.44)^a^	1 (0.38)^a^
Posthitis	3 (0.9)^a^	5 (1.09)^a^	1 (0.38)^a^
Balanoposthitis	2 (0.6)^a^	7 (1.53)^a^	0 (0)^b^
Paraphimosis	8 (2.4)^a^	6 (1.31)^a^	4 (1.54)^a^
Penile laceration	1 (0.3)^a^	5 (1.09)^a^	3 (1.15)^a^
Scrotal conditions			
Scrotal laceration	2 (0.6)^a^	7 (1.53)^a^	2 (0.76)^a^
Scrotal ectoparasitism	1 (0.3)^a^	0 (0)^a^	3 (1.15)^a^
Hydrocoele	0 (0)^a^	5 (1.09)^b^	0 (0)^a^
Hydrocoele with scrotal ectoparasitism	0 (0)^a^	0 (0)^a^	1 (0.38)^a^
Scrotal laceration with posthitis	0 (0)^a^	0 (0)^a^	1 (0.38)^a^
Scrotal laceration with balanitis	3 (0.9)^a^	0 (0)^b^	1 (0.38)^a^

Total	50 (15.06)^a^	86 (18.85)^a^	22 (8.46)^b^

^a,b^Significant at *P* < 0.05.
